# Alterations in cortical morphometry of the contralesional hemisphere in children, adolescents, and young adults with perinatal stroke

**DOI:** 10.1038/s41598-023-38185-8

**Published:** 2023-07-14

**Authors:** Karan Shinde, Brandon T. Craig, Jordan Hassett, Nomazulu Dlamini, Brian L. Brooks, Adam Kirton, Helen L. Carlson

**Affiliations:** 1 Department of Pediatrics, Cumming School of Medicine, University of Calgary, Calgary, AB Canada; 2 Calgary Pediatric Stroke Program, Alberta Children’s Hospital, 28 Oki Drive NW, Calgary, AB Canada; 3 Alberta Children’s Hospital Research Institute, Calgary, AB Canada; 4 Hotchkiss Brain Institute, University of Calgary, Calgary, AB Canada; 5 Neurosciences Program, Alberta Children’s Hospital, Calgary, AB Canada; 6 Department of Clinical Neurosciences, University of Calgary, Calgary, AB Canada; 7 Department of Psychology, University of Calgary, Calgary, AB Canada; 8 Department of Radiology, University of Calgary, Calgary, AB Canada; 9 Children’s Stroke Program, Hospital for Sick Children, Toronto, ON Canada; 10 Department of Pediatrics, University of Toronto, Toronto, ON Canada

**Keywords:** Neuroscience, Stroke, Neurology, Neonatal brain damage, Paediatric research

## Abstract

Perinatal stroke causes most hemiparetic cerebral palsy and cognitive dysfunction may co-occur. Compensatory developmental changes in the intact contralesional hemisphere may mediate residual function and represent targets for neuromodulation. We used morphometry to explore cortical thickness, grey matter volume, gyrification, and sulcal depth of the contralesional hemisphere in children, adolescents, and young adults after perinatal stroke and explored associations with motor, attention, and executive function. Participants aged 6–20 years (N = 109, 63% male) with unilateral perinatal stroke underwent T1-weighted imaging. Participants had arterial ischemic stroke (AIS; n = 36), periventricular venous infarction (PVI; n = 37) or were controls (n = 36). Morphometry was performed using the Computational Anatomy Toolbox (CAT12). Group differences and associations with motor and executive function (in a smaller subsample) were assessed. Group comparisons revealed areas of lower cortical thickness in contralesional hemispheres in both AIS and PVI and greater gyrification in AIS compared to controls. Areas of greater grey matter volume and sulcal depth were also seen for AIS. The PVI group showed lower grey matter volume in cingulate cortex and less volume in precuneus relative to controls. No associations were found between morphometry metrics, motor, attention, and executive function. Cortical structure of the intact contralesional hemisphere is altered after perinatal stroke. Alterations in contralesional cortical morphometry shown in perinatal stroke may be associated with different mechanisms of damage or timing of early injury. Further investigations with larger samples are required to more thoroughly explore associations with motor and cognitive function.

## Introduction

Perinatal stroke is a focal vascular brain injury that occurs between 20 weeks gestation and the 28th postnatal day^1^. Lifetime risk of stroke is highest in the perinatal period and occurs in approximately 1:1100 live births^2^. Perinatal arterial ischemic strokes (AIS) involve an infarction of a cerebral artery near birth, most commonly the middle cerebral artery. Periventricular venous infarctions (PVI) occur in utero prior to 32–34 weeks, primarily affecting periventricular white matter^3^. Perinatal stroke is the leading cause of hemiparetic cerebral palsy (HCP)^4^ with AIS and PVI both commonly damaging components of the motor system, resulting in contralateral hemiparesis.

Perinatal strokes represent an ideal human model for investigating developmental plasticity in otherwise normal brains due to their well-defined timing and focality. Converging evidence from various pre-clinical and human studies have improved our understanding of the effects on development of the motor system following such injuries^4–7^. Models of early motor development following perinatal stroke explored in animal^8^ and human^9^ brains suggest that developmental plasticity of the intact contralesional motor cortex may influence motor function of the paretic limb and has potential as a therapeutic target. Such compensatory mechanisms after perinatal focal brain injury may occur through altered developmental organization of the contralesional hemisphere^9–11^ but this process is not well understood.

In addition to motor disabilities, challenges with cognitive function may also occur after perinatal stroke^4,12–14^. Evidence suggests that children have a higher risk than peers of a secondary attention-deficit hyperactivity disorder (ADHD) diagnosis^15,16^ and that symptoms may be prevalent in almost half of children after perinatal stroke^17,18^. Deficits in executive function, characterized by poor attentional control, cognitive flexibility and problem solving behaviour, have also been documented in children with perinatal stroke^4,12,13^. Deficits in attention and learning may have serious functional consequences but little is known regarding mechanisms, limiting the development of informed interventional strategies^13^.

Advanced neuroimaging has shed light on mechanisms of brain development after perinatal stroke^19^, including diffusion tensor imaging^20–22^, functional MRI^23^, and region of interest (ROI) based volumetric analyses^24,25^, in addition to other neurophysiology-based measures^26^. Structural imaging studies in PVI have quantified lower grey and white matter volumes in motor-associated regions of the ipsilesional cerebral cortex (primary motor and sensory cortices), though specific associations with motor function were not found^25^. Group differences in thalamic volumes have been found after perinatal stroke showing modest inverse associations with motor function. Most notably, larger contralesional thalamus volumes were associated with poorer motor function^24^. Cerebellum volumes have also been shown to associate with function, specifically larger ipsilesional cerebellar volumes were linked to better scores on a pediatric stroke outcome measure^27^. A recent exploration of the structural connectome of the contralesional hemisphere in children with perinatal stroke suggested widespread alterations in network topography and high inverse correlations with clinical function^20^. Graph theory metrics quantified network organization and resulting associations showed that higher global efficiency, assortativity, and hierarchical topology were all associated with poorer motor function on multiple motor outcome measures. These studies taken together have demonstrated compelling structure–function associations but how these are reflected in the structure of the cortical surface is unknown.

Advancements in structural neuroimaging analysis techniques have allowed for quantification of surface-based morphometric measures such as cortical thickness, gyrification (surface folding patterns of the cortex) and sulcal depth, potentially providing insight into the development and reorganization of the cerebral cortex. A recent morphometry study in children with neonatal AIS found reduced cortical thickness, surface area and cortical volume in widespread areas of the *ipsilesional* hemisphere associated with the vascular territory of the middle cerebral artery, and greater gyrification, surface area and cortical volume in some areas of the *contralesional* hemisphere (occipital areas, precuneus) relative to controls^28^. This raises the intriguing possibility of compensatory alterations occurring in the intact hemisphere after very early injury. A more heterogeneous unilateral cerebral palsy population showed altered morphometry in both hemispheres, the degree of which correlated with motor and executive function^29^. Specifically, contralesional cortical thickness was higher for motor-related regions (including primary and supplementary motor, somatosensory areas, and the parietal cortex) compared to controls. Greater contralesional cortical thickness has also been associated with enhanced sensory function after arm rehabilitation in adults with chronic stroke^30^. These findings present evidence of possible compensatory cortical reorganization in the contralesional hemisphere and further validate the importance of the contralesional hemisphere as a therapeutic target.

The current study explored cortical morphometry of the intact contralesional hemisphere in children, adolescents, and young adults with perinatal stroke and possible associations with motor and executive function. Investigation of the contralesional hemisphere was the primary focus to explore possible compensatory cortical reorganization and reduce challenges associated with accurate normalization and segmentation in the ipsilesional hemisphere due to large lesion heterogeneity. We hypothesized that both AIS and PVI groups would have greater cortical thickness, cortical volume, gyrification, and sulcal depth in motor and executive function associated regions of the contralesional hemisphere compared to typically developing controls (TDC). Due to differences in mechanism and timing of injury, we also hypothesized that AIS and PVI groups would show differences in patterns of contralesional morphometry.

## Results

Seven AIS, two PVI, and two control participants were excluded due to the presence of head motion that precluded morphometric analysis. The final morphometry sample included 109 participants (AIS N = 36, PVI N = 37, TDC N = 36). Of the AIS participants, 10 had epilepsy (10/36, 27.8%). A subset of stroke participants (N = 73) completed all motor assessments (N = 45/73, 62%), including the Assisting Hand Assessment (AHA)^31^ and, the Box and Blocks Test^32^ for the affected hand (BBTA) and unaffected hand (BBTU). One AIS participant completed the AHA but not the BBTA or BBTU. This participant had a low AHA score (27/100). One PVI participant completed the BBTU but not the AHA or BBTA. A subset of stroke participants had parent reports of cognitive function completed (N = 22/73, 30%), the Behavior Rating Inventory of Executive Function (BRIEF)^33^ and the Attention Deficit and Hyperactivity Disorder (ADHD) rating scale (ADHD-5)^34^.

Population demographics by group are displayed in Table 1. A chi-square test revealed no significant differences in sex distribution between groups. Mean age was not different between groups (H_2_ = 3.8, *p* = 0.15). There was a significant difference in total intracranial volume (TIV) between groups (F_2,70_ = 32.9, *p* < 0.001). Tukey post hoc tests revealed that TIV was higher in the TDC (TIV_TDC_ = 1622.6 ± 167.5, *p* < 0.001) and PVI groups (TIV_PVI_ = 1537.8 ± 141.0, *p* < 0.001) compared to AIS (TIV_AIS_ = 1349.7 ± 132.8).

**Table 1:** Demographics of sample.

Demographics	AIS (N = 36)	PVI (N = 37)	All stroke (N = 73)	Controls (N = 36)
Sex				
Male	N = 22 [61%]	N = 24 [65%]	N = 46 [63%]	N = 23 [64%]
Age (SD) [min–max]	11.7 (3.9) [6.3–19.0]	11.0 (2.8) [6.6–19.7]	11.3 (3.4) [6.3–19.7]	12.3 (3.0) [6.5–19.0]
Side of stroke (MRI)				
Left (%)	23 (64%)	24 (65%)	47 (64%)	–
Size of stroke (cm^3^)	40.8 (38.7) [0.27–161]	–	–	–
TIV (mm^3^) (SD)	1349.7 (132.8)	1537.8 (141.0)	1445.0 (165.8)	1622.6 (167.5)
Motor outcomes (mean (SD))				
AHA (logit score)	57.4 (18.6) N = 22	65.0 (11.4) N = 24	61.3 (15.6) N = 46	–
BBTA (# of blocks)	24.5 (15.1) N = 21	29.7 (9.8) N = 24	27.3 (12.7) N = 45	–
BBTU (# of blocks)	52.3 (9.4) N = 21	51.7 (12.3) N = 25	52.0 (11.0) N = 46	–
Cognitive outcomes				
BRIEF mean T-score (SD)	N = 16	N = 6	N = 22	
Inhibit	52.9 (11.8)	55.5 (9.97)	53.6 (11.1)	–
Shift	58.9 (13.7)	58.8 (20.4)	58.9 (15.2)	–
Emotional control	60.8 (12.3)	56.7 (14.0)	59.6 (12.6)	–
Initiate	60.8 (16.1)	55.8 (15.0)	59.4 (15.6)	–
Working memory	62.6 (15.6)	56.5 (13.1)	60.9 (15.0)	–
Plan/organize	62.9 (15.4)	56.7 (12.4)	61.2 (14.6)	–
Organization of materials	54.3 (9.84)	51.7 (12.6)	53.5 (10.4)	–
Monitor	60.2 (13.4)	55.0 (13.2)	58.8 (13.3)	–
Behavioral regulation Index	58.7 (12.9)	57.5 (15.1)	58.4 (13.2)	–
Metacognition	62.6 (15.4)	56.7 (13.6)	61.0 (14.9)	–
Global executive composite	61.7 (14.3)	57.3 (14.7)	60.5 (14.2)	–
ADHD mean percentile (SD)	N = 16	N = 7	N = 23	–
Hyperactivity	56.4 (33.0)	68.0 (25.3)	59.9 (30.8)	–
Inattention	73.6 (27.0)	59.9 (29.6)	69.4 (27.9)	–
Total	68.9 (29.0)	65.4 (26.3)	67.8 (27.7)	–

*AIS* arterial ischemic stroke, *PVI* periventricular venous infarction, *SD* standard deviation, *MRI* magnetic resonance imaging confirmed lesion side, *TIV* Total intracranial volume, *AHA* assisting hand assessment, *BBTA* box and blocks affected, *BBTU* box and blocks unaffected, *ADHD* ADHD Rating Scale 5 (percentiles (SD)), *BRIEF* Behavior Rating Inventory of Executive Function (T-scores (SD)). The BRIEF and ADHD scales are negatively scored such that higher values represent poorer function.

For the subset of participants that had motor assessments (N = 45), age was not associated with assessments of motor function for the AIS group (all p-values > 0.1) and in the PVI group was positively associated with BBTA (r_s_ = 0.52, p = 0.009) and BBTU (r_s_ = 0.61, p = 0.001). There were no group differences between AIS and PVI groups for the AHA, BBTA, and BBTU.

For the subset of participants that had parent ratings of cognitive function (N = 22), age was not associated with ADHD ratings for either stroke group but was moderately associated with executive function for the AIS group on one subtest (Organization of materials: r = 0.59, p = 0.02). ADHD ratings were not significantly different between AIS and PVI groups (all p-values > 0.25). ADHD ratings for the inattention subscale and the total rating scale were significantly higher than the 50th percentile for the AIS group (Inattention W = 120.0, p = 0.008; Total W = 113.5, p = 0.02) but not the PVI group (all p-values > 0.11) suggesting higher parent-rated symptoms of ADHD in the AIS group than in an age- and sex-matched normative group. Four AIS but no PVI participants showed total ADHD rating scale scores over the 93rd percentile (~ 1 standard deviation (SD) above the mean, 2 females, 2 males, mean age (SD)[min–max] = 14.3(2.9)[10.2–16.4] years).

There were no significant differences in BRIEF score between AIS and PVI groups (all p-values > 0.39). Group BRIEF scores were higher than the expected T-score of 50 for most subtests in the AIS group but not the PVI group (Table 2) again suggesting higher parent ratings of executive function symptoms in the AIS compared to an age- and sex-matched normative group. Eight AIS but no PVI participants demonstrated mildly elevated BRIEF Global Executive Composite (GEC) scores (T-score > 60, 4 females, 4 males, age = 12.2(3.4)[7.2–16.4] years). Four AIS participants demonstrated clinically elevated BRIEF Global Executive Composite (GEC) scores (T-score > 70, 2 females, 2 males, age = 16.7(2.6)[13.3–19.0] years).

**Table 2:** Comparison between cognitive scores by stroke group and expected values.

Demographics	AIS	PVI
BRIEF	N = 16	N = 6
Inhibit	t_15_ = 1.0, p = 0.330	t_5_ = 1.4, p = 0.24
Shift	t_15_ = 2.6, p = 0.020*	t_5_ = 1.1, p = 0.34
Emotional control	t_15_ = 3.5, p = 0.003**	t_5_ = 1.2, p = 0.30
Initiate	t_15_ = 2.7, p = 0.017*	t_5_ = 1.0, p = 0.39
Working memory	t_15_ = 3.2, p = 0.006**	t_5_ = 1.2, p = 0.28
Plan/organize	t_15_ = 3.3, p = 0.004**	t_5_ = 1.3, p = 0.25
Organization of materials	t_15_ = 1.7, p = 0.104	t_5_ = 0.3, p = 0.76
Monitor	t_15_ = 3.0, p = 0.008**	t_5_ = 0.9, p = 0.40
Behavioral Regulation Index	t_15_ = 2.7, p = 0.017*	t_5_ = 1.2, p = 0.28
Metacognition	t_15_ = 3.3, p = 0.005**	t_5_ = 1.2, p = 0.28
Global Executive Composite	t_15_ = 3.3, p = 0.005**	t_5_ = 1.2, p = 0.28
ADHD	N = 16	N = 7
Hyperactivity	W = 76.0, p = 0.38	t_6_ = 1.9, p = 0.11
Inattention	W = 120.0, p = 0.008**	t_6_ = 0.9, p = 0.41
Total	W = 113.5, p = 0.02*	t_6_ = 1.6, p = 0.17

Group distributions were compared against expected values: BRIEF T-score = 50, ADHD percentile = 50. *p < 0.05, **p < 0.01.

### Cortical thickness

**Figure 1:**
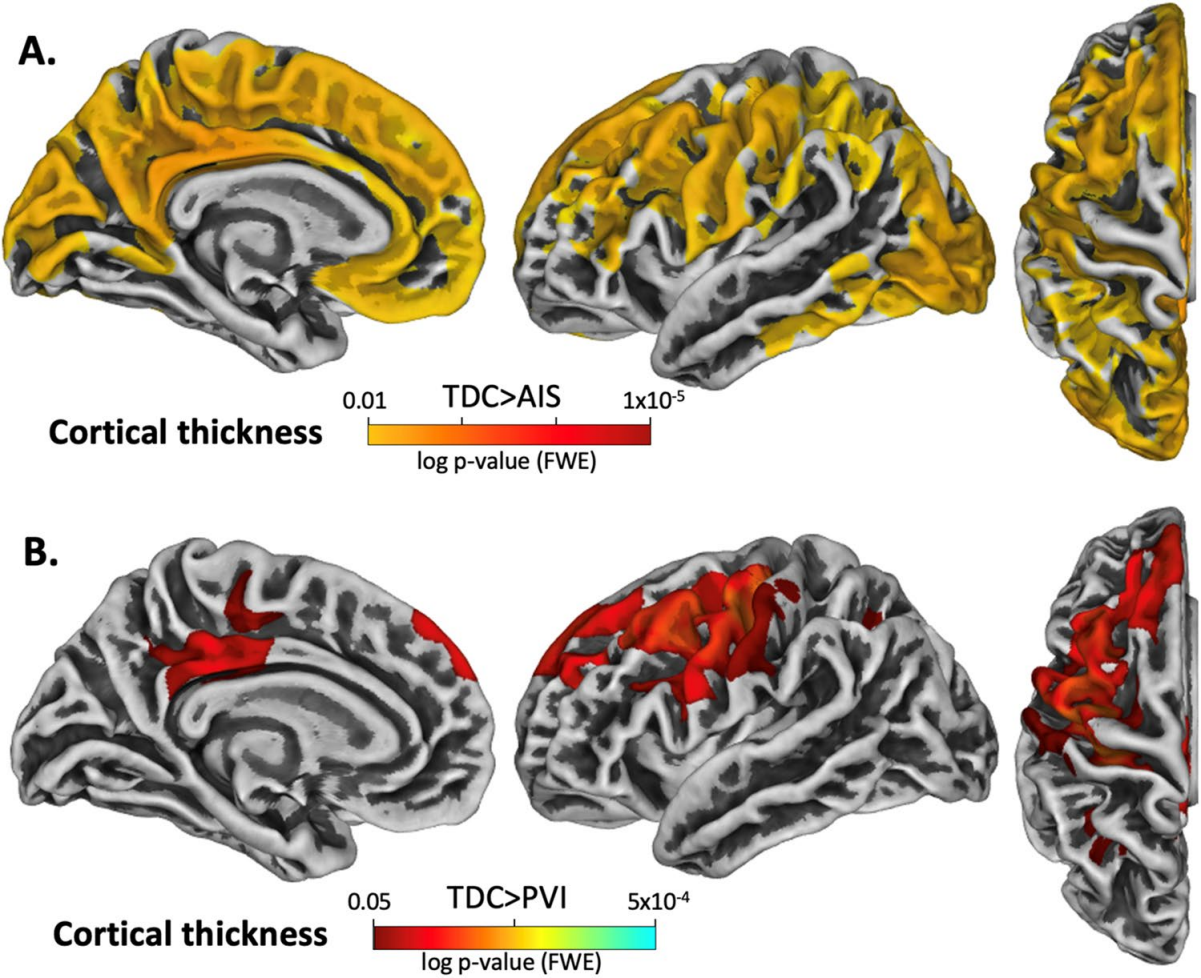
Illustration of significant group differences in cortical thickness for the contralesional hemisphere. The contrasts are displayed such that hot colours (yellow–red scale) denote areas where TDC had greater cortical thickness than AIS or PVI. (**A**) TDC > AIS, (**B**) TDC > PVI. Significance threshold was FWE corrected.

AIS participants exhibited significantly thinner cortex in several regions of the contralesional hemisphere in comparison to TDC. These included the medial wall, premotor and supplementary motor areas, frontal lobe, occipital lobe and within the lateral central sulcus (Fig. 1A). There were no areas showing thicker cortex in the AIS group compared to TDC. PVI participants also exhibited significantly thinner cortex in the contralesional hemisphere but within different regions, including the precuneus, posterior cingulate gyrus, superior frontal gyrus, lateral areas of the precentral gyrus, premotor areas, and superior parietal areas in comparison to TDC (Fig. 1B). No significant differences in cortical thickness were detected between PVI and AIS groups.

### Grey matter volume

**Figure 2:**
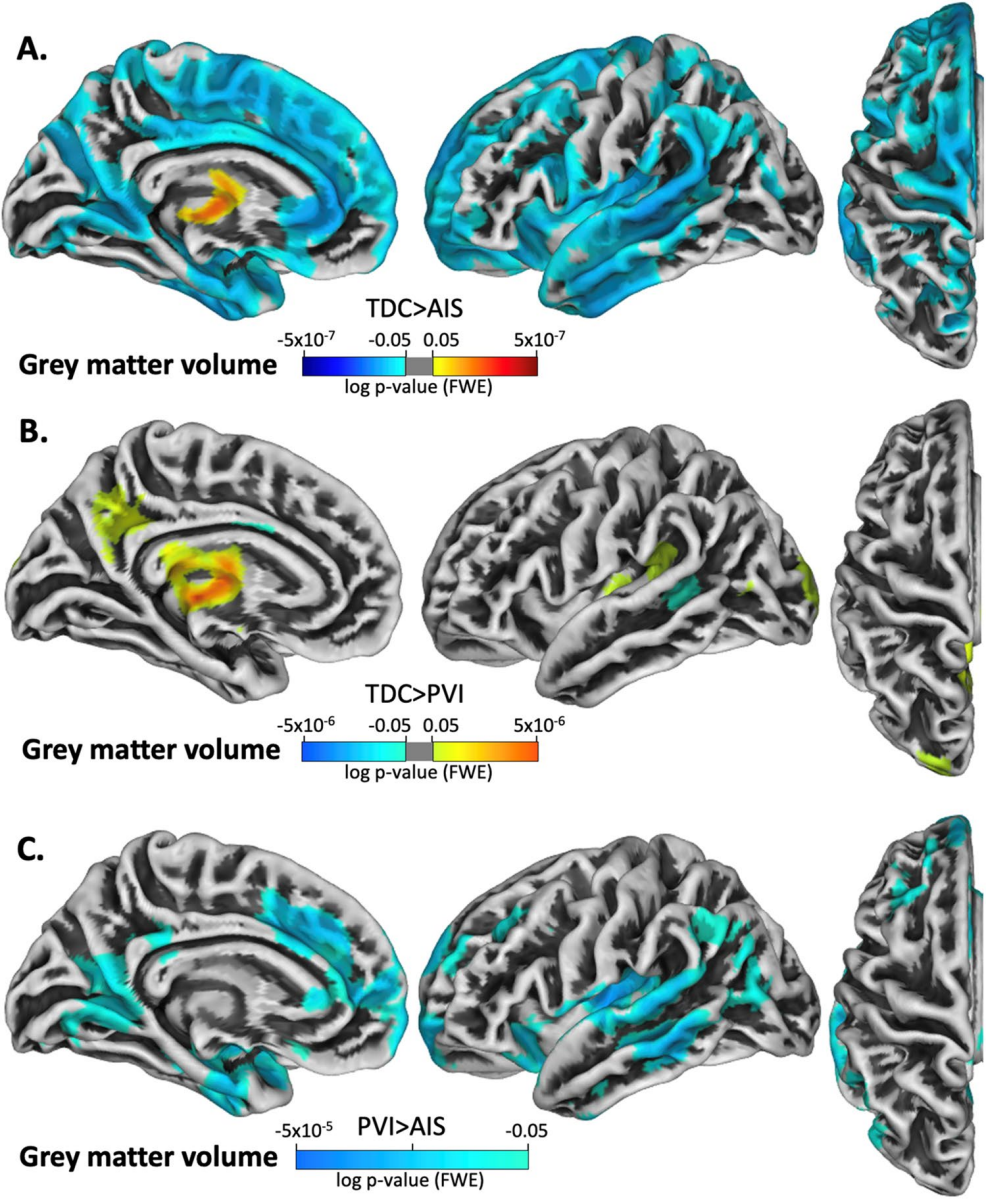
Projection of significant group differences in grey matter volume for the contralesional hemisphere. The contrasts are displayed such that hot colours (yellow–red scale) denote areas where the first group has greater grey matter volume than the second group. Cool colours (blue scale) denote areas where the second group has greater grey matter volume than the first group. (**A**) TDC > AIS, (**B**) TDC > PVI, (**C**) PVI > AIS. Significance threshold was FWE corrected.

AIS participants exhibited significantly greater grey matter volume relative to TDC within the contralesional precentral and postcentral gyri, anterior and posterior cingulate gyrus, posterior part of the isthmus cingulate, lingual cortex, superior temporal gyrus, precuneus, superior parietal gyrus, frontal pole and along the superior frontal gyrus (Fig. 2A). PVI participants exhibited significantly lower grey matter volume relative to TDC in the precuneus and superior temporal gyrus (Fig. 2B). The PVI group showed lower grey matter volume compared to AIS in the superior temporal gyrus, anterior cingulate, and the medial temporal lobe (Fig. 2C).

### Gyrification

**Figure 3:**
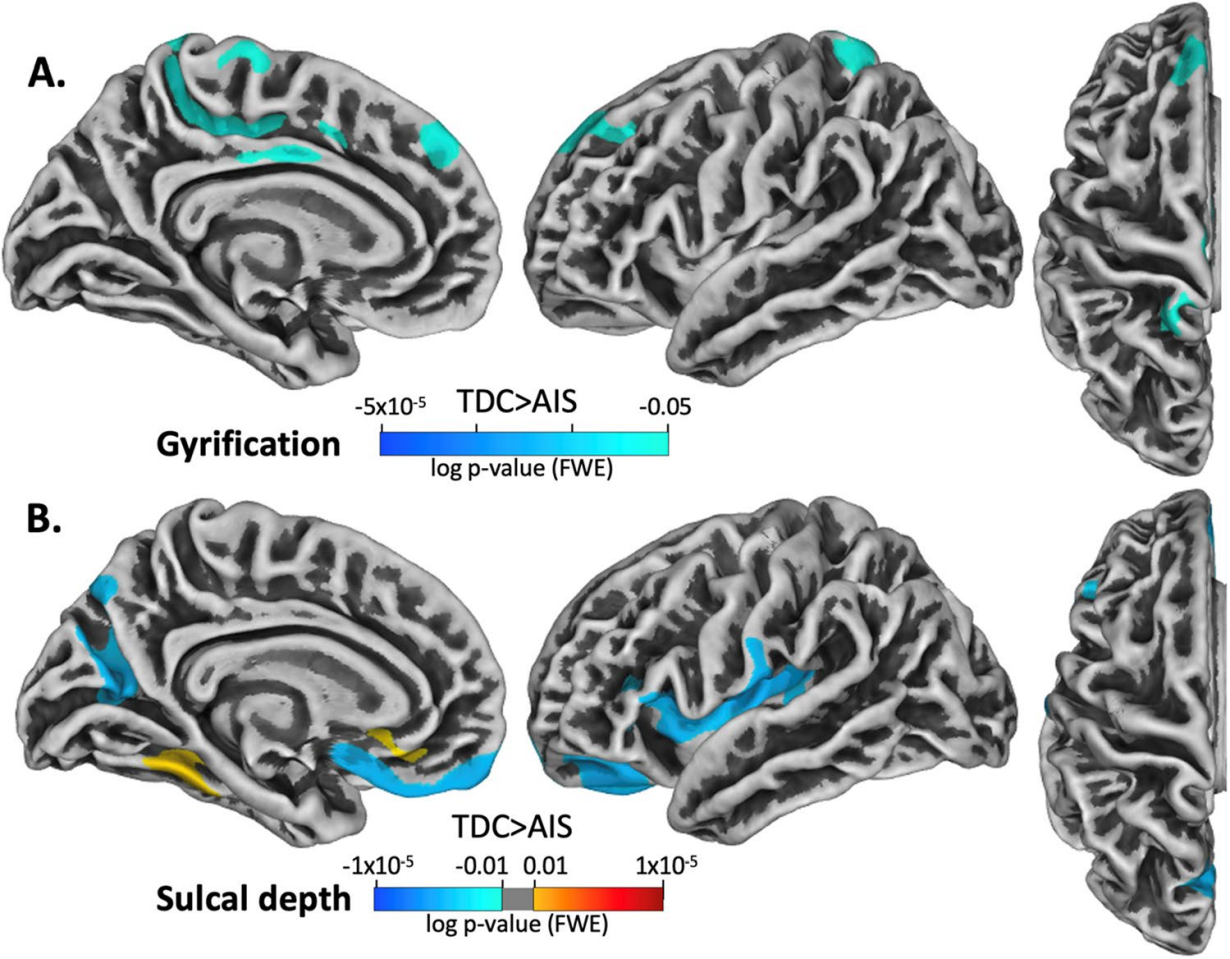
Projection of significant group differences in gyrification and sulcal depth for the contralesional hemisphere between TDC and AIS. The contrasts are displayed such that the hot colours (red-yellow scale) denote areas where TDC had greater gyrification values or sulcal depth than AIS and cool colours (blue scale) denote areas where TDC had lower gyrification values or lower sulcal depth than AIS. Significance threshold was FWE corrected.

AIS participants exhibited significantly greater gyrification relative to TDC within the middle cingulate gyrus, medial postcentral gyrus, and superior frontal gyrus (Fig. 3A). No significant differences in gyrification index were found between PVI vs TDC or between AIS vs PVI groups.

### Sulcal depth

Between-group comparisons revealed greater sulcal depth in AIS relative to TDC within the parieto-occipital fissure, inferior part of the medial orbitofrontal cortex, and the insula. AIS showed significantly lower sulcal depth in the superior part of the medial orbitofrontal cortex, and the fusiform gyrus (Fig. 3B). There were no significant differences in sulcal depth between PVI vs TDC or between AIS vs PVI groups.

### Correlations with clinical motor and executive function

There were no significant voxel-wise correlations between any of the cortical structure metrics and results from clinical motor, executive function, or ADHD assessments.

## Discussion

In this study, surface- and voxel-based morphometry approaches were used to investigate cortical structure differences in the intact contralesional hemispheres of children, adolescents, and young adults with perinatal stroke and HCP compared to typically developing peers. We present converging evidence that structural characteristics of the contralesional hemisphere are different, suggesting developmental neuroplastic changes following early unilateral injury. Specifically, compared to TDC, participants with AIS demonstrated lower cortical thickness, higher grey matter volume, higher gyrification, and higher sulcal depth values in areas within the contralesional hemisphere. Participants with PVI also showed lower cortical thickness and higher grey matter volume compared to TDC. Taken together, differences in multiple morphometry metrics suggest a higher surface area in multiple brain areas including those implicated in motor and cognitive function though no strong voxel-wise correlations were found between morphometry measures and clinical motor, executive function, or ADHD outcome scores.

Cortical thickness analysis revealed lower thickness in lateral premotor and supplementary motor areas, posterior cingulate gyrus, precuneus, and the frontal lobe near the superior frontal gyrus in both stroke groups relative to controls. Cortical thickness differences for the AIS vs TDC comparison appeared more widespread than those for the PVI vs TDC comparison, with cortical thickness differences in AIS vs TDC seen across most of the medial wall, supplementary motor areas, and a significant portion of the occipital lobe. Contrary to our original hypothesis of greater cortical thickness in contralesional motor areas, there were no areas of greater cortical thickness compared to controls within the more medial upper limb areas of primary motor or somatosensory cortices within the contralesional hemisphere for either stroke group. Lower cortical thickness was instead detected in more lateral motor and premotor areas. Greater cortical thickness was anticipated in motor areas of both stroke groups attributable to a shifting of function from ipsilesional into contralesional hemisphere motor areas, possibly related to the known persistence of ipsilateral corticospinal connections after early injury^9,10,26^. At birth, ipsilateral and contralateral projections from primary motor areas to lower motor neurons exist in approximately equal proportions^9^. Gradually, through the course of development in an activity-dependent manner, ipsilateral projections are pruned away to result in the typical contralateral representation found in adults with no neurological injuries^5^. In the case of perinatal stroke, this pruning process may not occur or may occur incompletely and because of differences in activity-dependent pruning, ipsilateral projects may persist^26^. Our results were not consistent with this original hypothesis although interestingly, thicker cortex has been demonstrated in the contralesional hemisphere motor areas in another study that was also associated with motor function^29^.

The absence of group differences in cortical thickness in these primary motor areas mediating upper limb function is somewhat surprising since we have previously found that motor and premotor areas show developmental alterations in both hemispheres after perinatal stroke. Specifically, using upper limb motor task fMRI (i.e., finger tapping) in a similar sample of children with perinatal stroke, we showed a greater amount of variability (compared to controls) in the location of primary motor cortex in both hemispheres as well as in the location of supplementary motor area^23^. However, it is likely that the reorganization of motor networks after injury is more complex than simple corticospinal laterality. A recent machine learning study investigated multiple imaging biomarkers and determined that more complex models of reorganization, including structural and functional connectivity of the wider motor network as well as connectivity of the basal ganglia, are more predictive of motor function in this population^35^.

Despite no group differences in cortical thickness for medial, upper limb motor-related areas in the stroke groups relative to TDC, group comparisons revealed large areas of higher grey matter volume in the medial precentral and postcentral gyri, posterior cingulate gyrus, precuneus, and along the superior frontal gyrus in the AIS group. The PVI group exhibited higher grey matter volume in smaller clusters located in the posterior temporal lobe, and lower volume in the precuneus and superior temporal gyrus relative to TDC. Assessment of the gyrification index revealed areas of greater gyrification in the middle cingulate gyrus, medial postcentral gyrus, and along the superior frontal gyrus in AIS when compared to TDC. These results indicate an inverse association between cortical thickness and gyrification, which is consistent with findings reported in past studies on healthy adults^36^. Why these differences were more notable in the AIS vs TDC comparison compared to the PVI vs TDC comparison is unknown but may relate to the larger cortical injuries in the lesioned hemisphere of AIS compared to the typically subcortical white matter injuries of PVI. The AIS group had ischemic injuries to their middle cerebral artery (MCA) and resulting tissue damage was localized to within the relatively large vascular territory perfused by the MCA. By contrast, the PVI group had tissue damage that was largely constrained to the periventricular white matter affected by the original venous infarction of the germinal matrix in utero. Differences in injury timing may also play a role in group differences in cortical morphometry, since AIS typically occurs near term in the first 28 days of life, whereas PVI occurs before 34 weeks of gestation^37^.

An additional intriguing possibility to explain group differences in morphometric patterns lies in tension-based cortical morphogenesis^38^. This theory suggests that mechanical tension in axons and dendrites within the brain drive changes in cortical folding and gyrification patterns seen in the cortex across development. Disruptions in the natural progression of such development, as may be seen in both AIS and PVI perinatal stroke, may explain group differences in cortical morphometry. Specifically, the primarily white matter injury seen in participants with PVI is very different than the more expansive cortical, subcortical and white matter injuries seen in the AIS group^37^. The earlier occurrence of PVI injuries (< 34 weeks gestation) compared to AIS (near term or in the first 28 days of life) is also consistent with an earlier disruption of cortical morphogenesis. Though more likely to affect morphometry in the injured hemisphere, such processes may also be disrupted in the intact hemisphere due to neuroplastic rewiring leading to the cortical morphometry differences seen here. Indeed, we have previously demonstrated transcallosal degeneration of contralesional structures^3,39^. A previous motor task fMRI study using transcranial magnetic stimulation (TMS) to probe corticospinal configurations supports this rewiring idea, showing ipsilateral control of the affected limb in both AIS and PVI groups^23^ as does an extensive TMS study demonstrating that ipsilateral and bilateral corticospinal configurations are relatively common in perinatal stroke and are associated with motor function^26^. Such a rewiring hypothesis is also consistent with the current finding that age was associated with motor performance, in the PVI group (BBTA and BBTU) though not the AIS group.

The presence of lower cortical thickness, but greater grey matter volume, gyrification, and sulcal depth we observed suggests a greater overall cortical surface area in regions of the contralesional hemisphere in stroke groups relative to TDC. Thinner cortex may reflect compensation allowing for crowding of more gyri into a smaller area. These findings agree with past studies^28^, suggesting that development of cortical gyrification is positively correlated with greater volume, but also operates under the constraint that keeping cortical thickness to a minimal level allows for more folding^36^. Higher cortical volume and gyrification in conjunction with lower cortical thickness in the primary motor and somatosensory cortices provides evidence in support of previously proposed mechanisms of reorganization and persistence of ipsilateral pathways in motor areas of the contralesional hemisphere after perinatal brain lesions^6,9,26^. It is possible that these regions in the contralesional hemisphere of individuals with perinatal stroke develop with more surface area to allow for clustering of cortical function into a reduced cortical area caused by limited function of the cortex in lesioned areas.

While direct voxel-based differences between AIS and PVI were seen only in grey matter volume, the spatial distributions of significant differences from TDC appeared different between the two stroke groups. This finding may again be related to the typically larger lesion sizes found in AIS participants compared to PVI, which may result in greater need for reorganization within both hemispheres during early development. If stroke-induced lesions are larger, more distant areas of the cortex may compensate, leading to larger morphometric changes and widespread variability among patients, which has been recently quantified using motor-task functional MRI^23^. AIS patients may also have more pronounced impairment of the affected limb in comparison to PVI, which suggests that activity-dependent learning may be different given different degrees of ability in these two groups.

We also did not see associations with clinical motor function and voxel-wise morphometry metrics in primary motor or sensory cortices. This finding suggests that specific, individual cortical areas may not independently mediate complex motor functions but rather are a component of a larger whole-brain motor network. Additionally, subcortical structures such as the basal ganglia^40,41^, the lesioned hemisphere, and the complex interplay between hemispheres, may also play a role in mediating residual motor function^35^, which is potentially consistent with the absence of voxel-wise correlations with clinical motor function we describe here.

Results showing consistent structural alterations in frontal and parietal areas were of interest given the higher incidence of ADHD and executive function symptomology in individuals with perinatal stroke compared to controls^12,13,42^. Group differences in morphometry metrics in the superior frontal gyrus, cingulate gyrus, dorsolateral prefrontal cortex, and precuneus in the AIS group compared to TDC suggest that attention networks may be impacted by the altered developmental trajectory observed in perinatal stroke, though the alterations are not constrained to only attention networks. These findings may be considered alongside results from past studies that revealed higher rates of ADHD diagnosis in participants with perinatal stroke in comparison to healthy controls^16,43^. Our current regression analyses between cortical structure metrics and cognitive scores showed no voxel-wise associations between executive function, ADHD, and cortical morphometry, a surprising finding given the brain areas where group differences were observed. Commonly reported executive function or attention deficits observed after perinatal stroke may not be associated with altered grey matter morphometry observed in widespread frontal areas or may have remained undetected due to limited statistical power in the relatively small sample of stroke participants with cognitive assessment scores.

We also found no statistical difference between the AIS and PVI groups for parent ratings of attention and executive function though more children with AIS were found to have clinically elevated scores and the mean for the AIS group was higher than a reference sample mean. It is fairly well established that children with AIS show poorer cognitive outcomes in addition to motor-related deficits whereas children with PVI typically have largely normal cognition^13^. These differences may occur for a number of reasons. Injury mechanism is very different in these two perinatal stroke types (arterial vs venous) and AIS lesions tend to be larger and involve more cortical and subcortical structures compared to the largely periventricular white matter injury seen in PVI. In our sample we had fewer children with PVI that had cognitive scores compared to AIS likely reflecting the higher referral rate for neuropsychological testing for AIS over PVI. This difference could also be due to lower statistical power to detect differences in the smaller PVI sample.

Several limitations must be acknowledged in the current study. Participants included ranged from 6 to < 20 years old, and therefore the sample represents a large range of developmental stages following early brain injury. The sample also excluded participants with higher levels of impairment due to the requirement to undergo a complex MRI protocol. This may have created some bias in the sample, limiting the applicability of these results in younger children and those with more severe motor and cognitive impairments. Furthermore, MRI scans of left sided strokes were reoriented, and brains were analyzed based on ipsilesional versus contralesional hemisphere. This was done to increase the power and sensitivity of the statistical analysis^44^ however innate differences in the left and right hemispheres of the brain may result in different developmental patterns between participants. In future larger studies, participants with left and right sided strokes could be analyzed separately, if the sample size permits, to determine whether structural alterations are similar among both groups. Sensitive white matter imaging techniques such as diffusion imaging and tractography may have shed additional light on specific white matter alterations but were not used in the current study. There remains the possibility that the significant group differences seen in medial areas were due to a midline shift secondary to atrophy in the ipsilesional hemisphere, however, participant scans were normalized to standard space and reviewed slice-by-slice for normalization accuracy to limit such effects. Lesioned brains also present many challenges to current neuroimaging software. Automated approaches to structural analysis are faster and more standardized but may be more susceptible to preprocessing errors occurring when processing lesioned brains^45^. Due to these challenges, this study focused on quantifying cortical structure alterations only in the intact contralesional hemisphere of perinatal stroke participants. A future direction for morphometry software development includes exploration of more powerful methods for neuroimaging analysis of lesioned brains, perhaps involving machine learning methods to optimize segmentation and normalization procedures. We also utilized images from two scanners which may have added additional variability to the sample, though scanner was used as a statistical covariate to minimize this issue. We did not explicitly screen the control group for preterm birth. Lastly, measures of motor, executive and attentional function were only collected for a subset of the stroke participants given that these assessments were part of clinical care or part of a separate rehabilitation trial. Ideally, motor and cognitive assessments would have been prospectively collected for all participants (including the TDC group) to increase power and enable direct comparisons among groups. The lack of association with cognitive assessment scores could additionally be due to limited statistical power in the current sample. Further cognitive domains could also have been investigated such as language, visual-spatial abilities, processing speed, memory and others that have been found to be disrupted in perinatal stroke. Lastly, we utilized parent report questionnaire measures of executive function and ADHD symptoms. While this afforded the largest dataset for our sample, direct and prospective measures of executive function and attention in the stroke participants themselves (as opposed to parent reports) may have provided a more sensitive measure of function.

## Conclusion

This study explored voxel-wise cortical morphometry in the intact contralesional hemisphere of children, adolescents, and young adults with unilateral perinatal stroke compared to typically-developing controls. We found widespread differences in cortical structure and volume in the contralesional hemisphere of the AIS group and moderate differences in the PVI group compared to TDC. These differences suggest an overall greater surface area in diverse areas of the contralesional hemisphere, which is generally consistent with past findings from related studies. Structural and volumetric alterations were found to be more pronounced in AIS relative to PVI, which is expected due to greater lesion size and increased severity of motor impairment in participants with AIS.

## Methods

This was a retrospective, cross-sectional, population-based study.

### Participants

Perinatal stroke participants were recruited from one of two sites; either via the Alberta Perinatal Stroke Project (APSP), a population-based research cohort^46^ or via the Hospital for Sick Children (SickKids) Children’s Stroke Program. The following inclusion criteria were applied at both sites: (1) MRI-confirmed, unilateral perinatal stroke syndrome according to previously validated criteria^3^ including AIS or PVI, and no evidence of diffuse, bilateral injury, history of epilepsy surgery, or additional neurological disorder (2) age 6 to < 20 years with term birth (> 36 weeks), (3) symptomatic hemiparesis [Pediatric Stroke Outcome Measure (PSOM) score > 0.5^47^ and perceived functional limitations by both child and parent], and (4) informed consent/assent.

Typically developing control (TDC) participants were recruited through a healthy controls recruitment program (HICCUP) based at the Alberta Children’s Hospital. TDC participants were aged 6 to < 20 years, right-handed, and had no self/parent-reported history of neurodevelopmental or psychiatric conditions and no MRI contraindications. TDC participants were similar in age (± 1 year) and sex to perinatal stroke participants.

Prior to participation, written informed parental consent and participant assent was obtained. The study was approved by the Conjoint Health Research Ethics Board at the University of Calgary and the Health Sciences Research Ethics Board at the University of Toronto. All research was performed in accordance with relevant guidelines/regulations at each site and in accordance with the Declaration of Helsinki.

### MRI data acquisition

T1-weighted anatomical images were obtained from two scanners. For the APSP cohort, MRI scans were obtained on a GE MR750w 3.0 Tesla scanner with a 32-channel head coil (GE Healthcare, Waukesha, WI). The three-dimensional (3D) fast spoiled gradient echo brain volume (FSPGR BRAVO) sequence included 166 contiguous axial slices (voxel size = 1 mm isotropic, matrix = 256 × 256, repetition time (TR) = 8.5 s, echo time (TE) = 3.2 ms). In the SickKids cohort, 3D Magnetization-Prepared Rapid Gradient-Echo (MPRAGE) images were obtained in the sagittal plane using a 3.0 Tesla Siemens Prisma scanner with a 20-channel head coil (192 contiguous slices, voxel size = 1 mm isotropic, matrix = 240 × 256, TR/TE = 2.3 s/3.0 ms).

### Preprocessing and cortical surface extraction

Prior to analysis, all scans were visually inspected slice-by-slice for artefacts arising from head motion such as ringing, blurring and striping^48^ and were excluded if such artefacts were detected. Unprocessed MRI scans for participants with left hemisphere strokes were reoriented in the x plane such that lesions were located on the right side to enable comparisons between the contralesional hemisphere in stroke participants and the dominant (left) hemisphere in TDC participants. Due to the heterogenous nature of lesion size, location, and hypothesized structural developmental changes, this study only focused on the contralesional hemisphere. Pre-processing of magnetic resonance (MR) images was conducted using automated procedures in the Computational Anatomy Toolbox (CAT12)^49^, an extension toolbox of Statistical Parametric Mapping software (SPM12) using MATLAB 2017b software (MathWorks, Natick, Massachusetts, USA). Images were bias-field corrected, skull stripped, and segmented into grey matter (GM), white matter (WM), and cerebrospinal fluid (CSF). Total intracranial volume (TIV) was calculated as a summation of GM, WM and CSF volumes in tissue class images in native space. A volume-based diffeomorphic DARTEL algorithm was applied to all surfaces for spherical registration to allow for inter-subject comparison^50^. After pre-processing, images were visually checked slice-by-slice for segmentation errors and normalization accuracy.

### Surface- and voxel-based morphometry

**Figure 4:**
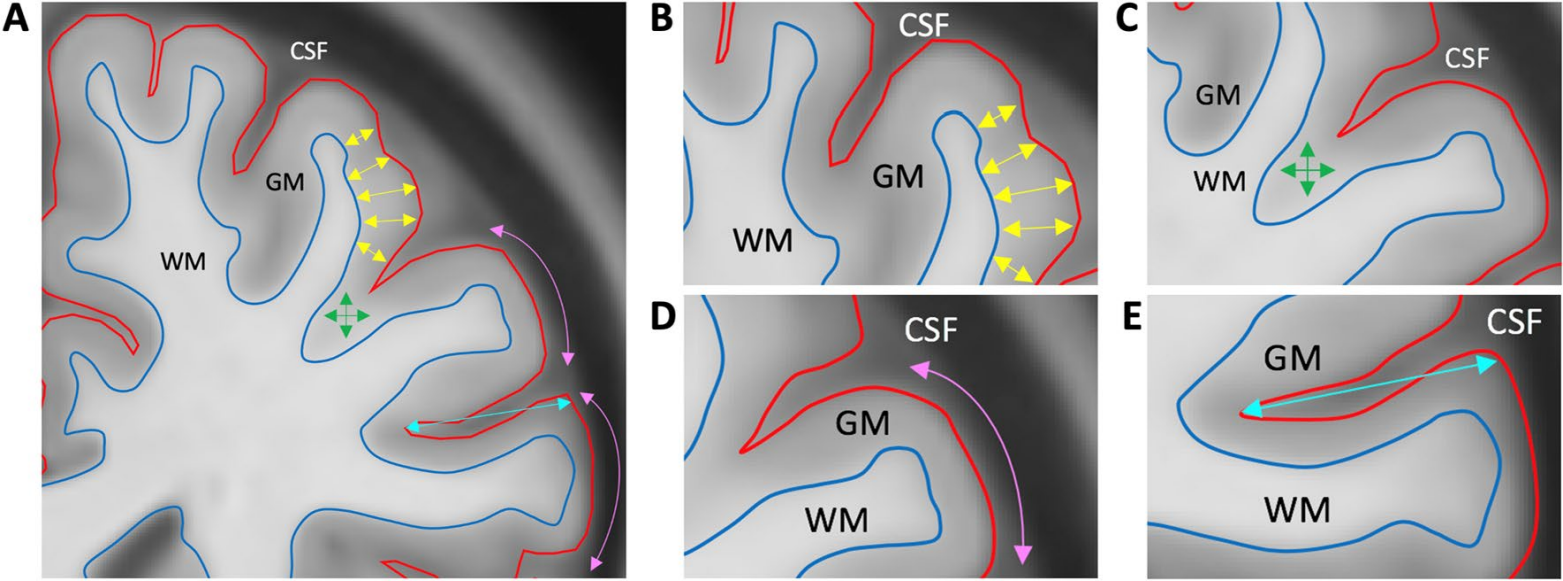
Cortical morphometry methods. (**A**) An illustration of surface and voxel-based morphometry variables on a coronal slice of a template image. (**B**) Cortical thickness (yellow arrows) was measured as the distance between the GM/WM boundary (blue) and the pial surface (red). (**C**) Grey matter volume (green) was measured via a voxel-wise comparison of tissue types. (**D**) Cortical gyrification (magenta) measured the degree of curvature of the pial surface. (**E**) Sulcal depth (cyan) is the maximal geodesic depth between the sulcal surface and the convex hull. *CSF* cerebrospinal fluid, *GM* grey matter, *WM* white matter.

A surface-based morphometry (SBM) voxel-based analysis approach was applied to quantify the primary imaging outcomes of cortical thickness, gyrification and sulcal depth (Fig. 4). CAT12 uses an automated voxel-based projection approach to compute cortical thickness based on distances between voxels at the white/grey matter boundary and the pial surface^49^. Gyrification index characterized cortical folding complexity by quantifying the ratio between the inner surface size and the outer surface size of the outer hull of the cortex based on an absolute mean curvature approach^51,52^. Sulcal depth maps were computed by first generating a convex hull surface from the pial surface, and then calculating the maximal geodesic depth between each vertex and its nearest vertex on the hull surface^53^. Voxel-based morphometry (VBM) analyses were computed to further explore volumetric characteristics via a voxel-wise comparison of tissue composition^54^. Finally, parametric maps for all measures were re-sampled and smoothed with a Gaussian heat kernel of 8 mm (FWHM)^55,56^.

### Clinical motor function

Clinical motor function of perinatal stroke participants was assessed using a series of validated motor outcome tests administered by experienced pediatric occupational therapists who were blinded to all clinical and imaging information. These included the Assisting Hand Assessment (AHA), and the Box and Blocks Test (BBT) for both the affected (BBTA) and unaffected (BBTU) upper extremities. The AHA is a play-based measure typically used to describe how children with a unilateral upper limb disability are able to use their affected limb during bimanual tasks^31^. The BBT corresponds to the total number of blocks participants can transfer from one box to another over a partition in 60 s^32^ and is used as a measure of unilateral gross manual dexterity. Higher scores on both assessments reflect better motor function.

### Executive function and attention

Executive function of perinatal stroke participants was assessed using the Behavior Rating Inventory of Executive Function (BRIEF), a parent questionnaire for evaluating executive function behaviours in children and adolescents^33^. All eight clinical subscales, as well as two broader indices (behavioral regulation and metacognition), and overall score (global executive composite) of the BRIEF evaluation were analyzed to explore associations with structural morphometry.

Attention of perinatal stroke participants was evaluated using the ADHD-5 rating scale for children and adolescents^34^ which is based on the diagnostic criteria for ADHD as described in the fifth edition of the *Diagnostic and Statistical Manual of Mental Disorders* (DSM-5). Scores for both subscales within the ADHD rating scale (inattention and hyperactivity-impulsivity) and a total scale were assessed.

Scores on both cognitive measures are negatively scored such that higher scores represent poorer performance. Scores reported here are percentiles (ADHD) or T-scores (BRIEF) calculated using large, age-matched normative groups for comparison.

### Statistical analysis

Shapiro–Wilk tests were used to determine the normality of data distributions. An independent samples Kruskal–Wallis test was used to assess differences in age between participant groups. A chi-square test determined differences in sex distribution. One-way analysis of variance (ANOVA) was used to evaluate group differences in TIV. Pearson and Spearman correlations explored associations between age, motor, and cognitive function scores. One-way analysis of co-variance (ANCOVA) with Bonferroni post-hoc correction was used to assess differences in scores for the AHA, BBTA, and BBTU assessments of motor function, with age included as a covariate. For cognitive scores, group differences between AIS and PVI were tested using a Student’s t-test. One-sample t-tests and Wilcoxon W tests examined whether cognitive scores were different from expected population values (BRIEF T-score = 50, ADHD percentile = 50) for each stroke group.

A general linear model (GLM) was used to assess morphometric differences among the three participant groups (AIS, PVI, TDC). Based on past studies revealing variations in brain volume with age^57^, age was included as a covariate in all statistical models. Two-tailed independent sample t-tests were performed between group pairs (AIS vs TDC, PVI vs TDC, AIS vs PVI) for each morphometric measure (cortical thickness, volume, gyrification index, and sulcal depth), with age and MRI scanner type included as covariates. For VBM analyses, TIV was also included as a covariate. Linear regression analyses were performed to explore voxel-wise associations between cortical surface measures and motor function (AHA, BBTA, BBTU) as well as cognitive function (BRIEF, ADHD) with age included as a covariate (and TIV for VBM analysis). As a non-parametric statistic, threshold-free cluster enhancement was applied with 10,000 permutations^58^. Family-wise error (FWE) correction (p_FWE_ < 0.05) was applied to resulting statistical maps at the cluster level to correct for multiple comparisons. Once the whole-brain analyses were complete, statistical maps for the non-lesioned hemisphere were visualized.

## Data Availability

Data will be available from the authors (helen.carlson@ahs.ca) upon reasonable request.
